# Role and regulatory mechanism of GPR37 in neurological diseases

**DOI:** 10.3389/fncel.2025.1617682

**Published:** 2025-07-31

**Authors:** Shuo Liu, Tao Bai, Xun Liu, Wenwen Zhao, Xin Li, Yi Sui, Juan Feng

**Affiliations:** ^1^The Fourth People’s Hospital of Shenyang, Shenyang, Liaoning, China; ^2^Department of Neurology, Shengjing Hospital of China Medical University, China Medical University, Shenyang, China

**Keywords:** GPR37, multiple sclerosis, autism spectrum disorder, pain, Parkinson’s disease, stroke

## Abstract

G protein-coupled receptor 37 is an orphan Class A GPCR predominantly expressed in the central nervous system (CNS), implicated in diverse physiological and pathological processes. This review summarizes current advances in the structural and functional understanding of GPR37, including its genomic localization, receptor architecture, endogenous ligands, and downstream signaling pathways. Emphasis is placed on its cell-type-specific expression across neurons, astrocytes, microglia, and oligodendrocytes, and how this expression dynamically shifts under pathological contexts such as Parkinson’s disease, stroke, and demyelinating disorders. GPR37 modulates neuroinflammatory responses, apoptosis, and oxidative stress through context-dependent mechanisms shaped by its ligands, including prosaposin, neuroprotectin D1, and osteocalcin. Additionally, GPR37 dysfunction–especially via receptor misfolding and ER stress–contributes to neuronal vulnerability. We further discuss its emerging role as a pharmacological target and potential biomarker in CNS disorders. By integrating findings across molecular, cellular, and disease models, we propose a context-dependent framework positioning GPR37 as a multifunctional regulator and therapeutic candidate in neurodegeneration.

## 1 Introduction

Neurological diseases are among the leading causes of disability and mortality worldwide, affecting not only the elderly but increasingly affecting younger populations (Neifert et al., 2020; Fang et al., 2022; Lin et al., 2023; Soto-Lara et al., 2023). These disorders encompass a broad range of conditions, including cerebral ischemia, multiple sclerosis (MS), spinal cord injury, neurodegeneration, and psychiatric illnesses (Alva-Díaz et al., 2020; Morello et al., 2020; Mishra and Rawal, 2023; Pagonabarraga et al., 2023; Dehghaniathar et al., 2025; Kühlein et al., 2025). Despite advances in diagnostic tools and disease classification, most neurological disorders remain poorly treated due to incomplete understanding of their pathophysiology.

G protein-coupled receptors, the largest family of membrane receptors in the human genome, play critical roles in neurotransmission, immune signaling, and neurovascular regulation (O’Callaghan et al., 2012; Guo P. et al., 2022; Öz-Arslan et al., 2024). Aberrations in GPCR signaling have been implicated in several brain disorders such as schizophrenia, depression, Huntington’s disease, and Parkinson’s disease (PD) (Hosford et al., 2018; Cao et al., 2023; Wong et al., 2023). Although over 800 GPCRs have been identified, only about 200 have been exploited pharmacologically, leaving a large number of “orphan GPCRs” receptors for which endogenous ligands remain undefined as potential untapped therapeutic targets (Maudsley et al., 2022; Zhao et al., 2025).

**FIGURE 1:**
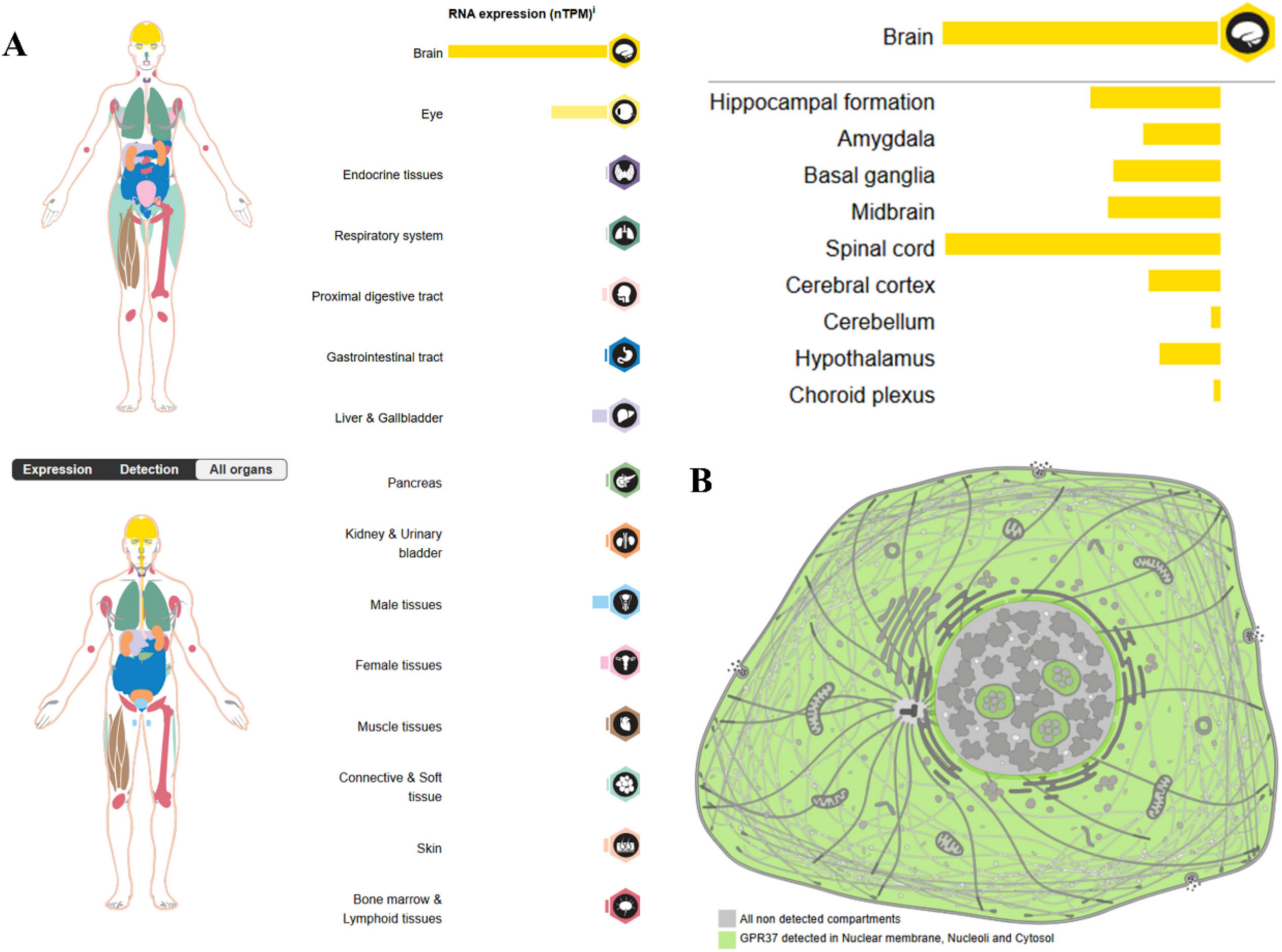
Expression of GPR37 in **(A)** human organs and **(B)** subcellular compartments (source: Human Protein Atlas). The most prominent expression of GPR37 is observed in the central nervous system, particularly in the brain and spinal cord. In contrast, its expression is relatively low in peripheral tissues such as the testis, placenta, and liver. This spatially distinct distribution pattern suggests that GPR37 may be involved in neurodevelopment, reproductive physiology, and metabolic regulation. At the subcellular level, GPR37 localizes to the nuclear membrane, nucleolus, and cytoplasm.

One such orphan receptor is G protein-coupled receptor 37 (GPR37), initially cloned from human brain cDNA libraries and classified as a member of the Class A (rhodopsin-like) subfamily of GPCRs (Liu et al., 2014). GPR37 is highly expressed in central nervous system (CNS) regions such as the substantia nigra, striatum, and hippocampus, as well as in glial and immune-related cells, including oligodendrocytes and microglia (Morató et al., 2021; Figure 1). These spatial expression patterns suggest that GPR37 may contribute to essential neurobiological functions and likely contributes to disease vulnerability.

In recent years, GPR37 has garnered increasing attention for its involvement in a variety of CNS disorders, including Parkinson’s disease, multiple sclerosis, stroke, glioma, autism spectrum disorder (ASD), and inflammatory pain (Wang et al., 2021; Guo D. et al., 2022; Zhang et al., 2024). Mechanistic studies have linked GPR37 to several biological processes, including dopaminergic regulation, myelination, immune modulation, and apoptosis. However, its complex and sometimes diverse and context-dependent roles across different cellular contexts remain a challenge for clinical translation.

This review aims to summarize the current understanding of GPR37 from a physiology-to-pathology perspective. We begin with its structural and molecular features, baseline functions, and ligand landscape, followed by the mechanisms of dysregulation and pathological involvement in various neurological disorders. Finally, we discuss emerging therapeutic strategies targeting GPR37 and highlight remaining challenges and opportunities.

## 2 Physiological role of GPR37

### 2.1 Structural and molecular overview

G protein-coupled receptor 37 is a member of the G protein-coupled receptor (GPCR) superfamily, specifically a Class A (rhodopsin-like) GPCR (Marazziti et al., 1997). GPCRs in this class typically possess a conserved seven-transmembrane (7-TM) α-helical domain architecture and are involved in a wide range of physiological functions, including neurotransmission and cellular signaling. Unlike many GPCRs with known ligands, GPR37 remains classified as an orphan receptor, as its full spectrum of endogenous ligands has not been definitively identified (Marazziti et al., 2001).

The GPR37 gene is located on human chromosome 7q31, a genomic region overlapping with autism susceptibility locus 1 (AUTS1), linked to neurodevelopmental disorders such as autism spectrum disorder (ASD) (Bonora et al., 2005; Fujita-Jimbo et al., 2012). This genetic colocalization has sparked interest in exploring whether GPR37 may play a role in disrupted neurodevelopmental processes in ASD. At the protein level, GPR37 consists of 613 amino acids and has an approximate molecular weight of 67.4 kDa (Lagerström and Schiöth, 2008). Like other Class A GPCRs, it comprises seven hydrophobic transmembrane domains connected by intracellular and extracellular loops. A distinctive feature of GPR37 is its unusually long extracellular N-terminal domain, which contains multiple consensus sequences for N-linked glycosylation and predicted cleavage sites (Zeng et al., 1997; Lawson and Wheatley, 2004). These post-translational modifications are believed to influence receptor folding, membrane trafficking, and ligand interaction, and may play roles in receptor aggregation or shedding under stress conditions.

Importantly, GPR37 shares structural homology with GPR37L1, a closely related orphan receptor that is predominantly expressed in glial cells. Although the two receptors exhibit approximately 35% sequence identity, especially within their transmembrane regions, they differ markedly in their N-terminal domains and expression profiles (Mukhaleva et al., 2024). GPR37 is highly expressed in neurons, especially in dopaminergic and oligodendroglial populations, while GPR37L1 is largely restricted to astrocytes and Bergmann glia. These distinctions in both structural and cellular context suggest functional divergence, although some overlapping signaling pathways have been proposed.

### 2.2 Expression patterns and cellular identity

G protein-coupled receptor 37 exhibits a complex and heterogeneous expression pattern across the central nervous system (CNS), reflecting its context-dependent roles in both neuronal and glial physiology. At the regional level, GPR37 is most abundantly expressed in the substantia nigra, striatum, hippocampus, cerebral cortex, and spinal cord (Liu et al., 2014; Morató et al., 2021). These regions are critically involved in motor control, learning and memory, and pain processing, consistent with GPR37’s putative roles in dopaminergic regulation, myelination, and neuroinflammatory signaling. Beyond tissue-level distribution, GPR37 shows marked cell-type specificity. It is predominantly expressed in neurons–particularly dopaminergic neurons–where it modulates synaptic function and stress responses (Morató et al., 2021). In addition to neurons, GPR37 is also found in oligodendrocytes, where it acts as a negative regulator of late-stage differentiation and myelin formation (Yang et al., 2016; Dierckx et al., 2022). Interestingly, under certain physiological and pathological conditions, GPR37 expression has been detected in astrocytes, although at lower levels compared to GPR37L1 (Ma et al., 2025). A recent single-cell transcriptomic analysis in a spinal cord injury model further demonstrated that both GPR37 and its ligand prosaposin are enriched in distinct glial subpopulations, indicating a dynamic and cell-state-specific expression profile (Wu et al., 2023). Subcellular localization studies have revealed that GPR37 can localize to the nuclear membrane, nucleolus, and cytoplasmic lysate, suggesting non-canonical roles in nuclear signaling and intracellular trafficking (Guo D. et al., 2022).

### 2.3 Baseline functions and endogenous ligands

In the absence of overt pathological stimuli, GPR37 contributes to neurophysiological homeostasis through a set of basal signaling mechanisms and ligand interactions. As an orphan GPCR, it has no clearly established endogenous ligand, but several candidates have been validated functionally in neuronal and glial models.

Prosaposin and its bioactive fragment, the 14-mer peptide TX14 (A), are among the best-characterized GPR37 ligands. These molecules activate GPR37 to promote neuroprotective effects, including enhanced cell survival, neurite outgrowth, and myelin maintenance (Jolivalt et al., 2006; Meyer et al., 2013). Mechanistically, PSAP/GPR37 engagement is associated with activation of the PI3K-Akt and ERK1/2 pathways, contributing to anti-apoptotic and anti-inflammatory responses in both neurons and oligodendrocytes (Bazan, 2005; Lukiw et al., 2005; Xu et al., 2013).

Another endogenous lipid-derived ligand, Neuroprotectin D1 (NPD1), also interacts with GPR37 and mediates anti-inflammatory and antioxidant effects. NPD1 stimulation of GPR37 suppresses pro-inflammatory cytokines and promotes cell survival via activation of PPARγ and downstream PI3K/Akt signaling cascades (Lukiw et al., 2005; Xu et al., 2013). This lipid-receptor axis suggests that GPR37 plays a protective role even under physiological conditions, functioning as a sensor and regulator of cellular stress and immune tone.

Osteocalcin, a peptide hormone traditionally associated with bone metabolism, has been implicated in GPR37-mediated neuromodulation. In developing brains, OCN was shown to cross the blood-brain barrier and influence hippocampal development and neurotransmitter synthesis through GPR37 (Rezgaoui et al., 2006; Gandía et al., 2013; Zhang and Wang, 2018). Moreover, OCN/GPR37 signaling contributes to the regulation of anxiety, cognition, and neuronal plasticity, highlighting a developmental and endocrine dimension to GPR37’s functionality (Qian et al., 2022).

In addition to these major ligands, several other candidates have been proposed. These include head activator (HA), aryl hydrocarbon receptor–related molecules (ARU), and chromogranin A (CGA), all of which have shown varying degrees of interaction with GPR37 *in vitro* or in cellular assays (Qu and Caterina, 2018; Bang et al., 2021; Zhang et al., 2022; Bolinger et al., 2023). While their *in vivo* relevance remains to be fully validated, these molecules expand the potential signaling landscape of GPR37 and suggest ligand versatility.

Collectively, these findings underscore GPR37’s ability to maintain CNS homeostasis through diverse endogenous ligands. By integrating peptidergic, lipid-based, and stress-associated signals, GPR37 functions as a molecular hub for neuroprotection, even in the absence of overt disease. Table 1 summarizes the known ligands and their functional consequences (Khoury et al., 2017; He et al., 2022).

**TABLE 1:** Studies of GPR37 in different neurological disorders.

Disease	Models	Major studies	Years	References
PD	PD patients	GPR37 (Pael-R) accumulation in Lewy bodies is associated with increased dopaminergic neuronal death.	2004	Murakami et al., 2004
PD	*Parkin*^–/–^ mice	Increased Pael-R expression is associated with a higher propensity for GPR37 misfolding and aggregation, ultimately leading to neuronal cell death.	2006	Kitao et al., 2007
PD	HEK293 cells and *Gpr37-*null mutant mice	GPR37 overexpression itself can induce cellular autophagy, which may prevent selective degeneration of GPR37-expressing neurons.	2009	Marazziti et al., 2009
PD	*Gpr37* mutant mice (Gpr37-KO)	In a series of cross-sectional studies analyzing the non-motor behaviors of adult and aged, male and female GPR37 KO mice and their wild-type littermates, it was found that aged GPR37 KO female mice exhibited mild improvements in olfactory function, whereas anxiety- and depression-like behaviors appeared to be significantly increased, demonstrating that deletion of the GPR37 receptor may exert neuroprotective effects in an age- and sex-dependent manner.	2013	Mandillo et al., 2013
PD	HEK293 and SH-SY5Ycells	It is demonstrated that GPR37 is exported from the endoplasmic reticulum in a normal manner after mature heterologous expression from HEK293 and SH-SY5Y cells, but its long extracellular N-terminus is subjected to limited protein hydrolysis between E167 and Q168 mediated by metalloproteinases.	2016	Mattila et al., 2016
PD	–	Discovery of significant PD gene networks and prediction of disruptive non-synonymous SNPs from selected gene pools using computer simulation methods.	2020	Rath et al., 2020
PD	PD patients	GPR37 is upregulated in sporadic PD and is a suitable potential PD biomarker.	2021	Morató et al., 2021
PD	PD patients	To determine whether GPR37 alterations can be used as a reliable biomarker for PD.	2024	Argerich et al., 2024
MS	GPR37-KO mice	Control of the transition from premyelinating oligodendrocytes to myelin-forming cells identifies GPR37 as a negative regulator of CNS myelin formation.	2016	Yang et al., 2016
MS	Gpr37/Gpr37L1 double knockout (DKO) mice	It was demonstrated that one of the most drastically reduced proteins in the brains of Gpr37/Gpr37L1 double knockout mice was the myelin-associated glycoprotein MAG, suggesting that GPR37 may be a potential drug target for the treatment of demyelinating diseases such as multiple sclerosis.	2017	Smith et al., 2017
Stroke	Gpr37-KO mice	Demonstration of the protective effect of GPR37 by modulating inflammatory and multicellular death pathways after ischemic stroke in mice.	2019	McCrary et al., 2019
Stroke	Gpr37^–/–^ mice	GPR37 is significantly upregulated after focal cortical infarction and functions as a key negative regulator of progenitor cell dynamics within the cerebral cortex after ischemic injury.	2021	Owino et al., 2021
ASD	ASD patients	GPR37 from autistic patients was analyzed to identify mutations associated with ASD.	2012	Fujita-Jimbo et al., 2012
ASD	ASD patients	ASD-related GPR37 mutations may lead to impairment of the CASPR2-MUPP1-GPR37 complex on dendrites associated with one of the pathogenic mechanisms of ASD.	2015	Tanabe et al., 2015
ASD	–	Discussion of genetic variants, relationship to core ASD behavioral deficits and identification of V1A, mGlu5, D2, 5-HT2A, CB1 and GPR37 as the best therapeutic targets among these OTRs.	2022	Annamneedi et al., 2023
Inflammatory pain	GPR37-KO mice	GPR37 activation in macrophages enhances phagocytosis and shifts cytokine release toward anti-inflammatory properties, thereby contributing to the reversal of inflammatory pain.	2018	Qu and Caterina, 2018
Inflammatory pain	GPR37-KO mice	Reveals a previously unrecognized role for GPR37 in regulating MΦ phagocytosis and inflammatory pain regression.	2018	Bang et al., 2018
Inflammatory pain	OCN^–/–^ mice	OCN is protective against LPS-induced acute inflammation by activating GPR37 in macrophages.	2022	Qian et al., 2022
Glioma	U251 cells	To investigate the effect of GPR37 upregulation on the proliferation of human glioma U251 cells.	2018	Zhang and Wang, 2018
Glioma	–	To investigate the relationship between GPR37 gene expression and clinicopathological factors of gliomas, patient prognosis, tumor infiltrating immune cell profile GSEA and methylation levels.	2023	Liang et al., 2023

## 3 GPR37 dysregulation and mechanistic disruption

### 3.1 Aggregation, cleavage, and receptor misfolding

G protein-coupled receptor 37 was originally identified as a substrate of the Parkinson’s disease-related E3 ubiquitin ligase Parkin and was therefore termed parkin-associated endothelin-like receptor (Pael-R) (Imai et al., 2001). In its misfolded or aggregated state, GPR37 tends to accumulate within the endoplasmic reticulum (ER), especially when ubiquitin–proteasome degradation is compromised. This accumulation initiates substantial ER stress and activates the unfolded protein response (UPR) (Mattila et al., 2016). Such a process is particularly detrimental in dopaminergic neurons of the substantia nigra, which are intrinsically vulnerable to proteostatic imbalance in Parkinson’s disease (PD).

The cellular consequences of GPR37 accumulation include ER swelling, disrupted calcium homeostasis, and up-regulation of canonical ER-stress markers such as GRP78 and CHOP (Marazziti et al., 2009). These stress responses ultimately lead to the activation of downstream pro-apoptotic cascades, including caspase-3 cleavage and mitochondrial dysfunction, contributing to dopaminergic neuronal loss.

In parallel with these aggregation-induced stress responses, GPR37 undergoes proteolytic cleavage at its extracellular N-terminus, producing a truncated form termed Ecto-GPR37 (Marazziti et al., 2009). This cleavage is mediated by the metalloprotease ADAM10 and releases soluble Ecto-GPR37 fragments into the cerebrospinal fluid (CSF), suggesting its potential as a biomarker for neurodegenerative disease (Lin et al., 2021). Notably, the generation of Ecto-GPR37 appears to be enhanced under pathological conditions, and emerging evidence indicates that it likely possesses distinct biological activities, although these remain incompletely understood.

Recent studies further indicate that GPR37 aggregation and cleavage are mechanistically linked, forming a convergent pathway that amplifies neuronal stress and degeneration. Truncated GPR37 has been shown to enhance CHOP expression and caspase-3 activation, exacerbating ER stress and promoting neuronal apoptosis in PD models (Lin et al., 2021). Taken together, both the intact misfolded receptor and its cleaved ectodomain fragment contribute to a molecular cascade of proteostasis failure and neurotoxicity, placing GPR37 at a critical intersection of receptor misprocessing and disease progression.

### 3.2 Pathway-level disruption

Beyond protein misfolding, GPR37 dysfunction extends to disruption of key intracellular signaling cascades, most notably the PI3K-Akt and MAPK-ERK pathways. These pathways are central to neuronal survival, glial homeostasis, and synaptic plasticity, and their dysregulation is a hallmark of multiple neurological conditions.

In healthy settings, GPR37 activation–particularly by neuroprotective ligands such as prosaposin (PSAP) or TX14A–leads to enhanced Akt phosphorylation, promoting cell survival and inhibiting apoptotic signaling. However, studies have shown that under pathological stress or when GPR37 is misfolded, this protective PI3K-Akt axis becomes compromised. For instance, GPR37-deficient mice exhibit increased vulnerability to MPTP-induced dopaminergic toxicity, a phenomenon linked to reduced Akt activity and weakened glial support (Jolly et al., 2018; Kunihiro et al., 2020). Moreover, GPR37 overexpression in certain stress models results in paradoxical inhibition of Akt signaling. This suggests that dysregulation arises not only from receptor loss-of-function but also from toxic gain-of-function (Kitamura et al., 2023).

In addition to PI3K-Akt, parallel disruptions have been observed in the MAPK-ERK pathway. This signaling axis is essential for myelination and synaptic remodeling, both of which are regulated by GPR37 in oligodendrocytes and neurons. In inflammatory models, impaired GPR37 function correlates with attenuated ERK1/2 phosphorylation, reduced oligodendrocyte precursor maturation, and exacerbated white matter damage (Song et al., 2005; Marazziti et al., 2011). Conversely, some evidence suggests hyperactivation of the ERK cascade in GPR37-overexpressing models, raising the possibility of context-dependent bidirectional dysregulation (Huang et al., 2014).

A critical caveat in interpreting these findings is the high homology between GPR37 and its paralog, GPR37L1. Many experimental tools, including antibodies and gene silencing constructs, may not sufficiently discriminate between the two receptors (Li X. et al., 2017). This is particularly problematic because GPR37L1 exhibits distinct, often opposing functions–being predominantly expressed in astrocytes and known to inhibit rather than activate the same pathways. Some studies have inadvertently conflated data between GPR37 and GPR37L1, necessitating caution in attribution of signaling outcomes (Liu et al., 2018).

Ligand specificity further complicates interpretation. Several putative GPR37 agonists–including TX14A, NPD1, and osteocalcin–have been shown to interact with other GPCRs or exhibit pleiotropic effects in different cell types. For example, while TX14A is considered a high-affinity ligand for GPR37, it also binds to other receptors under certain conditions, particularly when used at micromolar concentrations *in vitro* (Nguyen et al., 2020). A recent biophysical profiling study confirmed that TX14A exhibits partial promiscuity and suggested that its functional readout may depend on receptor conformation and membrane context (Breitwieser et al., 2024).

Such complexities highlight the importance of using precise genetic models and orthogonal validation techniques when dissecting GPR37 signaling. Knock-in systems with tagged endogenous GPR37, CRISPR-based isoform-specific knockouts, and single-cell RNA-seq paired with phospho-proteomics may help clarify how the receptor signals *in vivo* and how it signaling is perturbed in disease.

## 4 Disease manifestations of GPR37 dysregulation

### 4.1 Parkinson’s disease

Parkinson’s disease is a neurodegenerative disorder marked by progressive loss of dopaminergic neurons in the substantia nigra pars compacta (SNpc) and accumulation of Lewy bodies containing misfolded α-synuclein (Rocha et al., 2018; Riaz et al., 2024). GPR37, highly expressed in dopaminergic systems, has been implicated in PD pathogenesis through its involvement in protein misfolding, ER stress, and dopaminergic signaling.

Early postmortem studies revealed that GPR37, particularly in its misfolded and ubiquitinated forms, is enriched in a subset of Lewy bodies in sporadic PD patients (Huang et al., 2022). This receptor possesses a long N-terminal extracellular domain that is prone to misfolding during maturation in the ER. Under physiological conditions, the E3 ubiquitin ligase Parkin targets misfolded GPR37 for proteasomal degradation (Murakami et al., 2004). However, in Parkin-deficient neurons, GPR37 accumulates and forms insoluble aggregates, which co-localize with α-synuclein (Kitao et al., 2007; Yoon et al., 2020). Such accumulation induces ER stress and activates downstream apoptotic signals via PERK and CHOP pathways (Murakami et al., 2004; Leinartaité and Svenningsson, 2017). In animal models, overexpression of GPR37 exacerbates neuronal vulnerability to mitochondrial toxins such as MPTP, while its deletion confers resistance (Marazziti et al., 2009). This suggests a toxic gain-of-function phenotype when GPR37 is not properly degraded. These effects are more pronounced in the presence of defective Parkin, highlighting a pathogenic GPR37–Parkin axis. GPR37 also regulates dopaminergic neurotransmission beyond ER-associated stress. It forms heteromeric complexes with dopamine D2 receptors (D2R) in neuronal membranes and modulates receptor sensitivity and downstream signaling (Marazziti et al., 2009). Gpr37-knockout mice display exaggerated locomotor responses to acute D2R agonists and reduced tolerance to repeated stimulation, reflecting impaired D2R desensitization (Mandillo et al., 2013). GPR37 also interacts with the dopamine transporter (DAT), affecting dopamine uptake and synaptic DA availability. Its deletion results in increased DAT surface expression and altered DA reuptake dynamics (Marazziti et al., 2009). Genetic studies support GPR37’s relevance to PD risk. Rare functional variants in GPR37 have been identified in PD cohorts, with some mutations promoting receptor misfolding and increased ER stress responses *in vitro* (Rath et al., 2020). While these variants remain uncommon, their consistent association with protein quality control dysfunction aligns with the receptor’s mechanistic role in PD. Beyond its pathogenic implications, GPR37 shows translational potential as a biomarker. A cleaved extracellular fragment, termed ecto-GPR37, is elevated in the cerebrospinal fluid (CSF) of PD patients (Morató et al., 2021). In cross-sectional analyses, CSF ecto-GPR37 levels correlate with motor severity and distinguish PD from healthy controls and atypical Parkinsonism (Argerich et al., 2024). The presence of this fragment suggests both receptor cleavage and surface shedding, and its quantification may reflect disease progression.

### 4.2 Multiple sclerosis and demyelination

Multiple sclerosis is a chronic demyelinating disease of the central nervous system (CNS), marked by inflammatory episodes, myelin sheath loss, and incomplete remyelination. While immune responses have been extensively studied, accumulating evidence highlights intrinsic oligodendroglial mechanisms that shape the extent of injury and repair (Binamé et al., 2021). Among them, GPR37 has been identified as a critical regulator of oligodendrocyte maturation, with dual roles in developmental myelination and pathological demyelination (Morató et al., 2021). In both immune-mediated and toxin-induced demyelination models, GPR37 expression is downregulated during active disease phases. In the experimental autoimmune encephalomyelitis (EAE) model, which mimics MS-like neuroinflammation, GPR37 mRNA and protein levels are markedly reduced in spinal cord white matter at the peak of clinical symptoms, paralleling extensive myelin loss and axonal damage (Yang et al., 2016). Similarly, in toxin-based models such as cuprizone and lysolecithin, which induce demyelination without adaptive immunity, GPR37 downregulation persists, suggesting that its suppression is not solely driven by inflammation (Smith et al., 2017). The functional consequences of GPR37 loss have been characterized using Gpr37-knockout mice. Upon cuprizone exposure, Gpr37-deficient animals exhibit greater demyelination in the corpus callosum, poorer motor performance, and a pronounced delay in remyelination compared to wild-type controls (Dierckx et al., 2022). Proteomic analysis further reveals a significant decrease in myelin-associated glycoprotein (MAG) in knockout mice, with modest reductions in other structural proteins such as myelin basic protein (MBP) and proteolipid protein (PLP) (Smith et al., 2017). These findings suggest that GPR37 is necessary not only for preserving myelin integrity under stress but also for effective repair once damage has occurred. At the cellular level, GPR37 appears to influence the timing and efficiency of oligodendrocyte precursor cell (OPC) differentiation. *In vitro*, activation of GPR37 by its endogenous ligand prosaptide suppresses cAMP production and inhibits the EPAC–Raf–ERK1/2 signaling cascade, arresting OPCs in a pre-myelinating stage characterized by high Nkx2.2 and low MBP expression (Wu et al., 2023). Loss of GPR37 removes this developmental brake, accelerating differentiation and leading to premature myelination. However, the resulting myelin sheaths tend to be disorganized and morphologically aberrant when co-cultured with neurons (Schmidt et al., 2024). These data support a model in which GPR37 functions as a molecular timer, fine-tuning the pace of OPC maturation to ensure orderly myelin wrapping during development. Transcriptionally, Gpr37 expression is dynamically regulated during oligodendroglial maturation. It is selectively upregulated at the late OPC stage under the control of transcription factors such as Sox10 and the co-activator Zfp488 (Qian et al., 2021). This stage-specific expression pattern implies that GPR37 acts transiently to limit terminal differentiation until appropriate environmental or developmental cues are received. Beyond cell-autonomous effects, GPR37 modulates the glial microenvironment in demyelinating conditions. In cuprizone-treated Gpr37-knockout mice, microglia adopt a more pro-inflammatory phenotype, producing higher levels of TNF-α and IL-1β, while secreting less IL-10 (Ma et al., 2025). These cytokine shifts correspond with slower clearance of myelin debris and impaired recruitment of OPCs to lesion sites. Notably, GPR37 activation in oligodendrocytes promotes the release of IL-6, which in turn dampens microglial activation, forming a negative feedback loop between damaged neurons, glia, and immune cells (An et al., 2021). This bidirectional communication reflects the receptor’s broader role in orchestrating multicellular responses during myelin injury and recovery.

Although human data remain limited, single-nucleus RNA sequencing from chronic MS lesions has identified GPR37-enriched oligodendrocyte subsets bordering demyelinated plaques (Dierckx et al., 2022). Immunostaining of post-mortem MS tissues also reveals GPR37-positive cells localized to peri-lesional areas, though overall expression appears diminished relative to non-lesioned white matter (Smith et al., 2017). These spatial patterns mirror findings from animal models and suggest that GPR37 may contribute to lesion containment and endogenous repair.

### 4.3 Stroke and post-ischemic repair

Stroke leads to long-term disability, featuring acute ischemic injury followed by chronic inflammation and incomplete repair. Recent evidence has implicated GPR37 in both acute injury responses and the regulation of neurogenesis and tissue remodeling during post-ischemic recovery.

In animal models of cerebral ischemia, GPR37 expression is upregulated in the penumbral cortex and hippocampus within 48–72 h after stroke onset, with predominant localization in neuronal populations and neural progenitor cells (NPCs) (Wang H. et al., 2025). This spatial and temporal pattern suggests a role for GPR37 in the endogenous repair program rather than the initial ischemic cascade. Immunohistochemical analyses demonstrate GPR37 colocalization with doublecortin (DCX)-positive NPCs in the dentate gyrus, as well as NeuN-positive mature neurons, indicating its potential involvement in both neuroprotection and neuroregeneration. Functionally, loss-of-function studies have demonstrated that GPR37 facilitates neural progenitor cell survival, proliferation, and migration following stroke. Gpr37-knockout mice subjected to middle cerebral artery occlusion (MCAO) exhibit reduced numbers of BrdU + /DCX + cells in the subgranular zone of the dentate gyrus and the subventricular zone, suggesting impaired NPC expansion and mobilization (Mouhi et al., 2022). This defect is accompanied by enlarged infarct volumes, higher rates of neuronal apoptosis, and worse functional recovery, implicating GPR37 in both cytoprotection and endogenous repair. Mechanistically, GPR37 appears to regulate these processes via the mTOR signaling pathway, which is a critical node linking nutrient sensing, cellular survival, and regeneration. In post-stroke brain tissue, GPR37 deficiency leads to downregulation of phosphorylated mTOR and its downstream effectors S6 kinase and 4E-BP1, particularly in regions enriched for NPCs (McCrary et al., 2019). This signaling impairment correlates with reduced neuronal survival and impaired structural plasticity, including fewer dendritic spines and thinner dendritic arbors in peri-infarct cortex. In contrast, pharmacological activation of GPR37 by prosaptide restores mTOR pathway activity and promotes NPC proliferation and neurite extension *in vitro* and *in vivo*, suggesting a ligand-sensitive mechanism of repair modulation (Owino et al., 2021).

These findings highlight GPR37 as a key modulator of stroke recovery, bridging acute injury and neurogenic regeneration. Unlike many protective pathways that are restricted to the early injury window, GPR37 expression persists throughout the subacute and chronic phases, aligning it temporally with neurogenic and plasticity-promoting processes. This makes it an attractive target for post-stroke interventions aimed at enhancing endogenous brain repair.

### 4.4 Autism spectrum disorder

Autism spectrum disorder comprises a continuum of neurodevelopmental conditions defined by social-communication deficits and restricted, repetitive behaviors. Linkage analyses first pointed to chromosome 7q31-33–termed AUTS1–as a susceptibility region, and GPR37 lies squarely within that locus (Bonora et al., 2005). Two independent cohorts subsequently identified rare, conserved amino-acid substitutions in GPR37: T589M and R558Q. Both variants segregated with autism in multiplex families and reside in intracellular loops that influence receptor trafficking (Fujita-Jimbo et al., 2012). Functional assays revealed that R558Q reduces cell-surface delivery of GPR37, promotes retention in the endoplasmic reticulum, and triggers mild ER-stress, mirroring the misfolding-prone behavior already linked to Parkinsonian pathology. Notably, the variant’s impact extends beyond folding: in cultured hippocampal neurons, R558Q fails to accumulate in distal dendrites, leading to simplified dendritic arbor and reduced spine density (Tanabe et al., 2015). Molecular interactome studies provide a mechanistic context for these phenotypes. GPR37 forms a postsynaptic scaffold with CASPR2–a synaptic cell-adhesion molecule encoded by CNTNAP2 and itself an ASD gene–and the multi-PDZ protein MUPP1. Disruption of this CASPR2/MUPP1/GPR37 complex by the R558Q mutation weakens PDZ-dependent clustering at excitatory synapses and alters miniature excitatory postsynaptic current frequency (Tanabe et al., 2015). Given that mutations in CASPR2 or MUPP1 independently cause social deficits and epilepsy, these data place GPR37 within a convergent synaptic-adhesion network whose perturbation may underlie a subset of ASD cases.

G protein-coupled receptor 37’s influence extends to dopaminergic circuits implicated in stereotypic behaviors. Co-immunoprecipitation and functional uptake assays show that wild-type GPR37 associates with the dopamine transporter (DAT) and negatively regulates its surface density. Knock-down of GPR37 elevates DAT-mediated uptake, lowers extracellular dopamine, and augments amphetamine-induced locomotion–phenotypes reminiscent of hyper-dopaminergic mouse models that display repetitive self-grooming (Tabuchi et al., 2007). These findings align with human imaging studies reporting dopamine-signal imbalance in ASD and suggest that loss-of-function GPR37 variants could exacerbate stereotypies via dysregulated DAT trafficking.

Network-level analyses reinforce GPR37’s involvement in ASD. A recent review of twenty-three GPCRs genetically associated with ASD highlighted GPR37, alongside oxytocin (OXTR) and metabotropic glutamate (mGluR5) receptors, as a “high-yield” therapeutic target because its signaling pathways intersect both social and repetitive behavior modules (Annamneedi et al., 2023). Network modeling identifies GPR37 as a hub linking neuropeptides and classical neurotransmitters, consistent with its dual role at excitatory synapses and within dopaminergic axons. Although coding variants in GPR37 are rare in the general population, their observed penetrance in specific familial cases suggests that they may contribute meaningfully to disease phenotypes. *In vitro* studies using patient-derived induced pluripotent stem-cell (iPSC) neurons carrying the R558Q variant have reproduced ER stress and dendritic defects. Treatment with pharmacological chaperones such as 4-phenyl-butyrate has been shown to partially restore surface trafficking, spine density, and neuronal firing synchrony, suggesting modulation of mutant receptor function (Fujita-Jimbo et al., 2012; Bian et al., 2024).

Whether GPR37 ectodomain shedding also occurs in ASD remains unclear and might serve as a peripheral biomarker. Serum samples from a pilot cohort of autistic children showed a non-significant trend toward elevated soluble GPR37 fragments, but larger studies with age-matched controls are needed to clarify diagnostic value (Annamneedi et al., 2023).

### 4.5 Glioma and tumor biology

Glioblastoma multiforme, the most aggressive form of glioma, exhibits rapid proliferation, extensive infiltration, and strong resistance to therapy. GPR37 has emerged as a molecule involved in both tumor cell–intrinsic pathways and immunological alterations in the tumor microenvironment.

At the cellular level, GPR37 is upregulated in high-grade gliomas compared to adjacent normal tissues, as shown by transcriptomic and immunohistochemical analyses (Liang et al., 2023). Overexpression of GPR37 in U251 glioma cells promotes proliferation, migration, and colony formation, while silencing GPR37 via siRNA attenuates these tumorigenic behaviors (Zhang and Wang, 2018). Mechanistic studies further reveal that GPR37 activates the PI3K/Akt signaling pathway, increasing levels of phosphorylated Akt (p-Akt). Knockdown of GPR37 leads to p-Akt reduction, G1 phase arrest, and upregulation of the cyclin-dependent kinase inhibitor p21, linking GPR37 to oncogenic signaling and cell cycle progression in glioma cells (Bian et al., 2024). Beyond tumor cell-autonomous functions, GPR37 also influences the glioma immune microenvironment. *In silico* deconvolution of GBM transcriptomes reveals a positive correlation between GPR37 expression and infiltration by immunosuppressive myeloid cells, including tumor-associated macrophages (TAMs) and M2-like microglia (Liang et al., 2023). This is consistent with prior findings showing that GPR37 modulates microglial polarization toward an M2 anti-inflammatory phenotype (Liu et al., 2014). The enrichment of M2-polarized immune cells in GPR37-high tumors suggests that the receptor facilitates immune evasion, enabling tumor cells to grow in a permissive microenvironment. The convergence of these two functional domains–tumor proliferation and immune suppression–underscores GPR37’s multifaceted contribution to glioma biology. Unlike traditional oncogenes limited to intracellular signaling, GPR37 may act as a dual regulator, enhancing both intrinsic cell growth capacity and extrinsic immune modulation. This duality opens avenues for therapeutic targeting: inhibiting GPR37 could theoretically slow tumor progression while reshaping the immunological niche. However, given GPR37’s neuroprotective and homeostatic roles in normal brain tissue, systemic inhibition may risk off-target effects. Future strategies may require isoform-specific inhibitors or local delivery methods that maximize tumor specificity. In sum, GPR37 integrates oncogenic signaling and immune landscape modulation in gliomas, positioning it as a promising–but complex–therapeutic target in malignant brain tumors.

### 4.6 Inflammatory and chronic pain

Chronic inflammatory pain arises from the complex interplay between nociceptive neurons and immune cells in peripheral and central compartments. Recent findings implicate GPR37 as a key regulator at the intersection of immune modulation and neural excitability, acting through both macrophage-mediated resolution programs and direct neuronal inhibition of pain transmission (Binamé et al., 2021). GPR37 is expressed in peripheral macrophages and contributes to the resolution of inflammatory pain by promoting a phagocytic and anti-inflammatory phenotype. Activation of GPR37 by ligands such as NPD1 and TX14A enhances macrophage efferocytosis, increases IL-10 production, and accelerates resolution of inflammation-associated pain states (Qu and Caterina, 2018; He et al., 2022; Qian et al., 2022). In contrast, Gpr37-deficient mice exhibit delayed clearance of apoptotic neutrophils, prolonged inflammation, and sustained mechanical hypersensitivity, indicating a critical role in pain termination rather than initiation. This function is distinct from classical pro-resolving pathways like resolvins or annexins, as GPR37 appears to gate the transition from inflammatory to recovery phase by modulating macrophage behavior.

At the level of nociceptive circuits, GPR37 is expressed in dorsal root ganglion (DRG) neurons and spinal cord dorsal horn neurons (Binamé et al., 2021). Activation of GPR37 reduces spontaneous excitatory postsynaptic currents (sEPSCs) in these neurons, thereby attenuating central sensitization and pain amplification. Electrophysiological recordings show that pharmacological stimulation of GPR37 significantly dampens synaptic transmission in pain pathways. Importantly, these effects are independent of peripheral inflammation and suggest a direct neuro-modulatory function of the receptor. In Gpr37 knockout mice, increased frequency and amplitude of sEPSCs correlate with enhanced nociceptive behaviors following peripheral injury, further underscoring the receptor’s dual action in both peripheral resolution and central transmission. Ligand specificity also plays an essential role in the function of GPR37 in pain modulation. NPD1, a neuroprotective docosanoid derived from DHA, binds to GPR37 and activates downstream pro-resolution pathways. Similarly, TX14A, a synthetic analog of prosaposin, triggers anti-inflammatory responses in both macrophages and DRG neurons via GPR37 activation (Qu and Caterina, 2018; He et al., 2022; Qian et al., 2022; Reinertsen et al., 2023). These ligands may represent endogenous protective signals released during tissue stress or damage, positioning GPR37 as a sentinel receptor responsive to inflammatory resolution cues.

**FIGURE 2:**
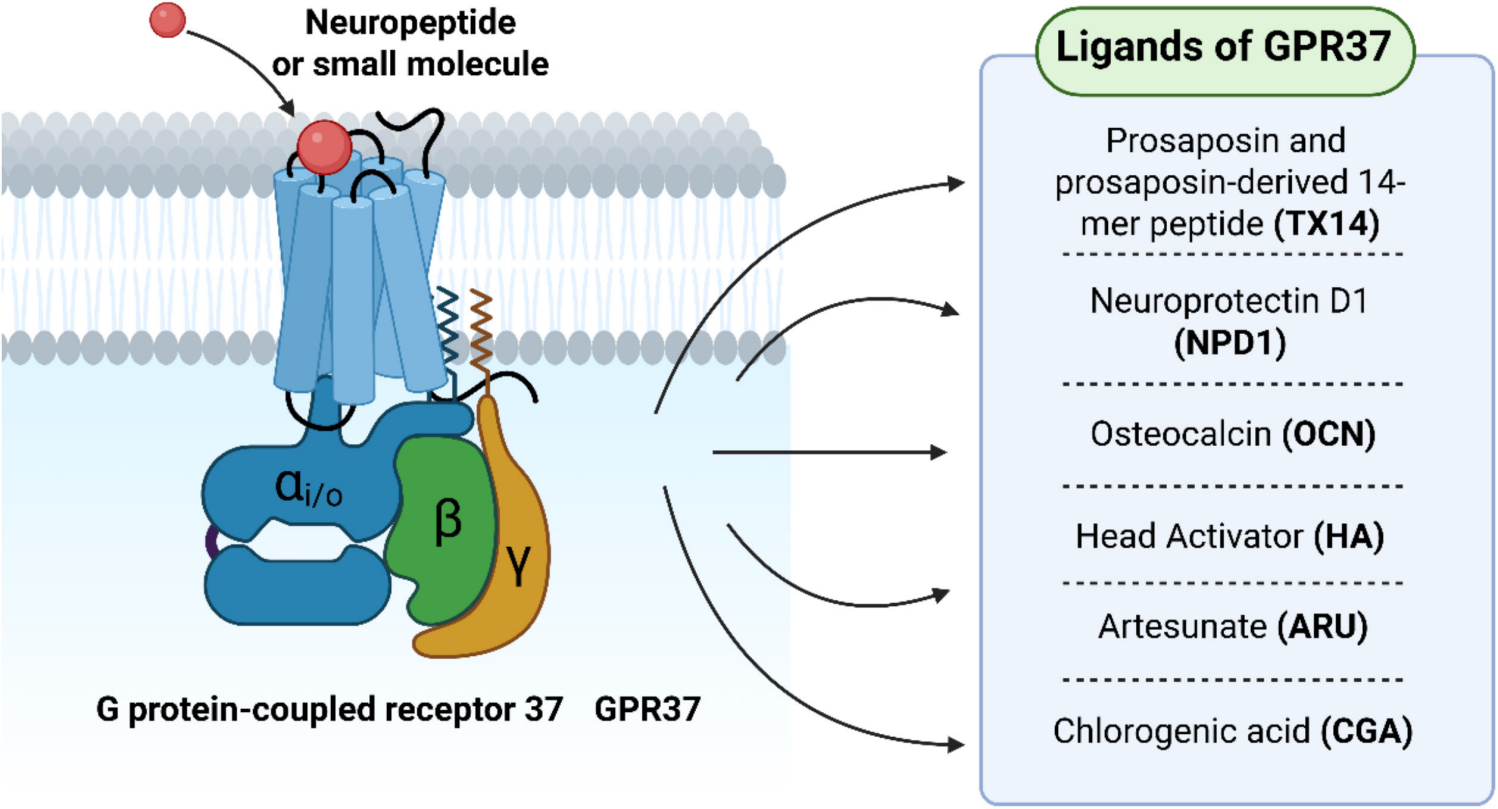
G protein-coupled receptor 37 (GPR37) regulates macrophage-mediated neuroinflammation and pain signaling. During tissue injury or inflammation, macrophages are recruited to the lesion site and polarize into pro-inflammatory (M1) or anti-inflammatory (M2) phenotypes. GPR37 modulates this polarization process and contributes to the resolution of neuroinflammatory pain responses.

In pathological contexts such as neuropathic pain, the receptor appears to act as a brake on neuroinflammation. In models of spared nerve injury and carrageenan-induced inflammation, Gpr37 knockout mice display prolonged pain responses and increased pro-inflammatory cytokine expression (Bang et al., 2018; Wang K. et al., 2025; Figure 2). Conversely, GPR37 activation enhances IL-10 expression and M2-like polarization in macrophages, reducing spinal cord glial activation and pain perception. This dual action–immune resolution and neuronal suppression–makes GPR37 a compelling target for chronic pain disorders, where both inflammation and central sensitization co-exist. GPR37’s role also intersects with spinal microglia and astrocytes, although expression in glial cells remains unclear. Some studies suggest indirect modulation through macrophage-neuron interactions or systemic anti-inflammatory feedback loops (Zhao et al., 2023). The lack of microglial GPR37 expression does not exclude downstream effects, especially as GPR37 signaling modulates IL-10, which in turn affects glial states and synaptic plasticity in pain circuits. Table 1 listed the studied roles of GPR37 in different neurological disorders.

## 5 Therapeutic strategies and translational perspectives

### 5.1 Ligand-based agonist strategies

**FIGURE 3:**
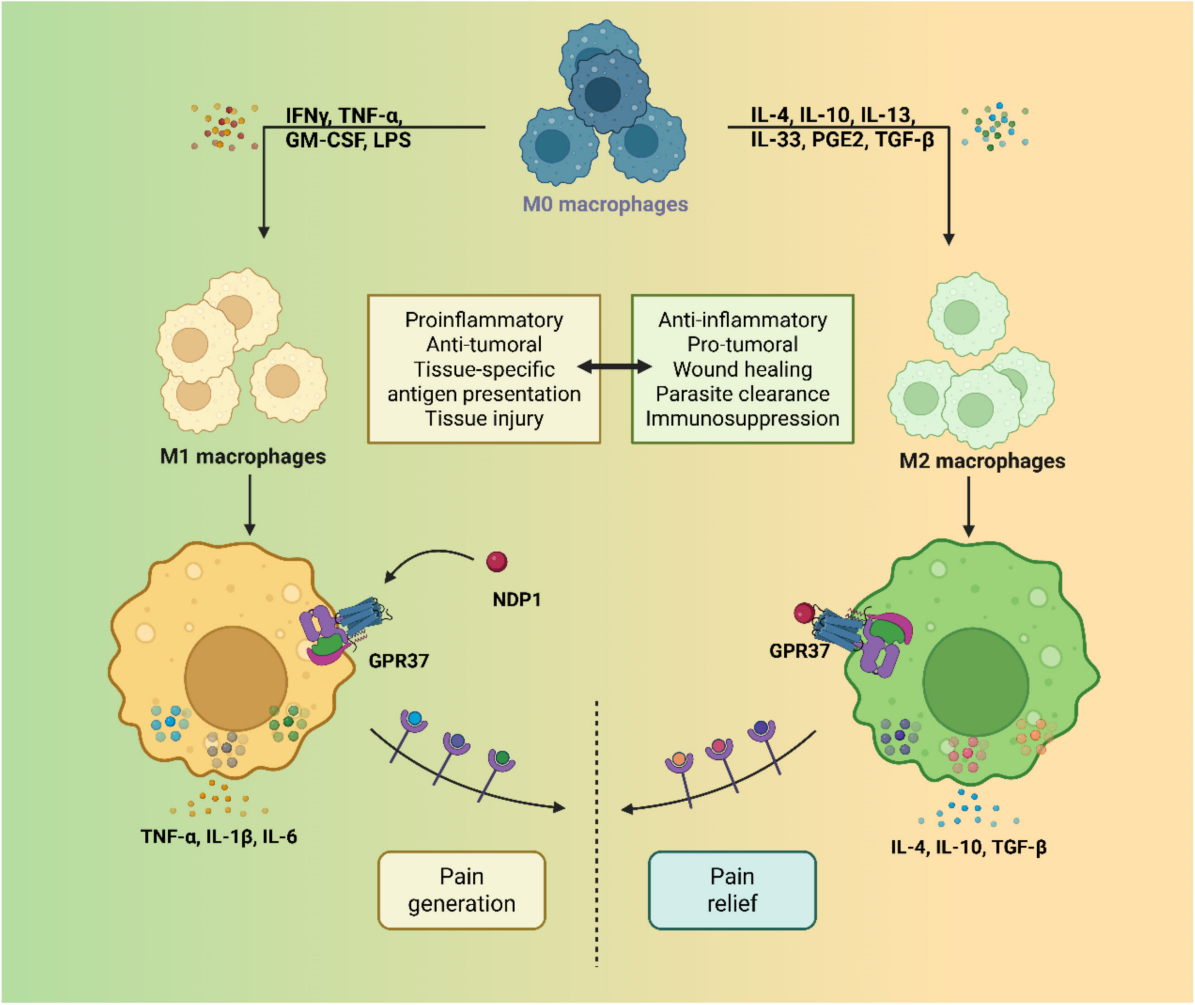
Structural diagram of GPR37 and its ligand-binding features. GPR37 comprises seven hydrophobic transmembrane α-helices that span the cell membrane to form a barrel-like conformation. This transmembrane architecture is critical for membrane anchoring and signal transduction upon ligand binding.

Therapeutic modulation of GPR37 has centered on identifying ligands capable of activating the receptor in a targeted and context-dependent manner. Among several candidates, TX14A, neuroprotectin D1 (NPD1), osteocalcin (OCN), and head activator (HA) have emerged as prominent agonists, each engaging distinct aspects of GPR37-mediated biology (Figure 3).

Prosaposin-derived 14-mer peptide A, a 14-mer peptide derived from the prosaposin protein, is one of the most well-characterized GPR37 agonists (Lukiw et al., 2005; Jolivalt et al., 2006; Meyer et al., 2013). It binds selectively to GPR37 and initiates neuroprotective signaling, particularly under stress conditions such as excitotoxicity or hypoxia. TX14A activates downstream pathways that reduce oxidative stress and stabilize mitochondrial function, contributing to cell survival in neural and glial populations. In animal models, TX14A administration has been shown to reduce infarct volume and protect dopaminergic neurons, indicating its potential in both ischemic and neurodegenerative contexts (Bazan, 2005; Meyer et al., 2013).

Neuroprotectin D1, a lipid-derived docosanoid generated from DHA, functions as an endogenous ligand of GPR37 with notable anti-inflammatory and neuroprotective effects (Rezgaoui et al., 2006; Xu et al., 2013; Zhang and Wang, 2018). NPD1 binding to GPR37 promotes IL-10 expression in macrophages and enhances phagocytosis of apoptotic cells, supporting tissue recovery in models of neuroinflammation. NPD1 also downregulates NF-κB activity and reduces cytokine release, indicating dual action on both immune and neural compartments. Importantly, Gpr37-deficient mice fail to respond to exogenous NPD1, affirming the ligand–receptor specificity.

Osteocalcin, traditionally associated with bone metabolism, has recently been identified as a peptide ligand for GPR37, linking peripheral signals to central nervous system function (Gandía et al., 2013; Bang et al., 2021; Qian et al., 2022; Bolinger et al., 2023). By activating GPR37 in a calcium-dependent manner, OCN modulates dopaminergic signaling and may aid in pain resolution. Some studies have suggested that OCN–GPR37 interaction may also influence synaptic plasticity, although its downstream signaling pathways in the brain remain underexplored. In preclinical pain models, systemic OCN delivery reduced mechanical hypersensitivity, an effect absents in Gpr37 knockout mice.

Head activator, a neuropeptide originally isolated from hydra, has also been implicated in GPR37 activation (Qu and Caterina, 2018; Zhang et al., 2022). Although its physiological relevance in mammals remains debated, *in vitro* studies suggest that HA can induce receptor internalization and ERK phosphorylation via GPR37. Its ability to activate downstream neurogenic or proliferative programs positions HA as a molecular probe for studying GPR37 signaling in neural stem cells and glioma models.

Collectively, these ligands exhibit diverse structural classes–ranging from peptides to lipids–and engage GPR37 through different mechanisms and tissue contexts. While TX14A and NPD1 primarily drive neuroprotection and immune resolution, OCN and HA illustrate the receptor’s integration with endocrine and regenerative signaling. Such diversity underscores the therapeutic flexibility of GPR37 as a target, allowing for disease-specific ligand tailoring.

Despite these advances, several challenges remain. First, ligand–receptor interactions have largely been characterized in rodent models, and translational pharmacology in human tissues is lacking. Second, ligand specificity remains an open question: GPR37 shares partial homology with its paralog GPR37L1, and cross-reactivity may confound *in vivo* outcomes. Third, the blood–brain barrier penetration of peptide ligands like TX14A and OCN remains suboptimal, prompting the need for delivery innovations. Table 2 listed GPR37-related ligands, sources, functions, major studies and references.

**TABLE 2:** GPR37 ligand information.

Ligands	Origin	Function	Major research	References
Prosaposin/TX14(A)	Derived from saposin C (a sphingolipid activating protein)	Shown to activate GPR37 in *in vitro* experiments, possibly involved in neuroprotection and myelin formation.	It is somewhat controversial and some studies have failed to reproduce its binding activity, which needs further validation.	Zhang et al., 2022
NPD1	In specific cells such as neurons and retinal pigment epithelial cells, DHA is converted to NPD1 by 15 - lipoxygenase.	May mediate apoptosis or inflammatory responses via GPR37, especially in dopaminergic neurons.	Preliminary experiments support their binding, but the mechanism has not been fully elucidated.	Qu and Caterina, 2018
OCN	Expressed and produced primarily by osteoblasts in vertebrates.	Regulation of lipid metabolism, insulin and glucose homeostasis, energy expenditure, vascular disease.	Several studies have shown that OCN binds specifically to GPR37.	Qian et al., 2022
HA	A neuropeptide found in worms, with possible analogs in mammals.	Promotes neurite growth and is neuroprotective.	Early studies suggested that HA might activate GPR37, but evidence for this was lacking.	Rezgaoui et al., 2006
ARU	A semi-synthetic derivative from *ArteMisia annua* L., a first-line antimalarial drug	It has anti-inflammatory effects, colitis, and rheumatoid arthritis in humans.	ARU treatment leads to a rapid decline in parasitemia by promoting phagocytosis-mediated clearance of parasite cells.	Khoury et al., 2017
CGA	It is widely found in fruits and vegetables and is the second most important ingredient in coffee beans.	Anti-inflammatory, antioxidant and anti-apoptotic effects.	Further studies are needed to elucidate the interaction between CGA and GPR37.	He et al., 2022

### 5.2 Genetic and pharmacological interventions

In addition to ligand-based strategies, genetic and pharmacological approaches targeting GPR37 offer complementary tools to dissect its role in CNS pathology and explore therapeutic potential. These methods include siRNA-mediated knockdown, gene knockout models, overexpression systems, and emerging small-molecule modulators. siRNA silencing of GPR37 has been employed in various *in vitro* systems to study its function in tumor biology and neuroinflammation. In glioma cell lines, transient knockdown of GPR37 using specific siRNA leads to reduced Akt phosphorylation and a concomitant increase in cell cycle inhibitors such as p21, resulting in G1 phase arrest (Yu et al., 2024). This supports a role for GPR37 in promoting cell proliferation through PI3K/Akt signaling. Similarly, in microglial cultures, Gpr37 silencing attenuates IL-10 release following stimulation, implicating its regulatory role in neuroimmune responses. Genetic knockout models provide a powerful platform for studying GPR37 functions *in vivo*. Gpr37−/− mice display heightened vulnerability to MPTP-induced dopaminergic neurodegeneration, impaired myelin repair in demyelination models, and exaggerated inflammatory responses in the CNS (Song et al., 2005). These phenotypes corroborate the receptor’s involvement in neuroprotection, oligodendrocyte maturation, and inflammation resolution. Importantly, behavioral assays reveal subtle motor and cognitive deficits in knockout animals even in the absence of overt pathology, suggesting a baseline physiological role for GPR37 in CNS homeostasis.

Conversely, overexpression systems have been instrumental in uncovering the potential toxic gain-of-function effects of GPR37 misregulation. In cellular models, overexpression of full-length GPR37 frequently leads to ER retention and activation of unfolded protein response (UPR) pathways, consistent with its role as Pael-R in Parkinson’s disease-related ER stress (Li L. et al., 2017). These findings highlight the need for balanced GPR37 expression to maintain proteostasis, where either loss or excess can be deleterious, depending on cellular context. In addition to genetic manipulation, pharmacological modulation of GPR37 remains an area of active investigation. Although highly selective small-molecule agonists or antagonists are not yet widely available, efforts have been made to screen compound libraries for modulators that alter GPR37 signaling without inducing aggregation or cytotoxicity (Bian et al., 2024). Such agents may prove valuable not only as therapeutic leads but also as research probes for dissecting GPR37-mediated pathways with temporal and dose precision.

### 5.3 Biomarker development and clinical potential

As mechanistic understanding of GPR37 deepens, interest has turned toward its utility as a biomarker in neurological disorders, particularly Parkinson’s disease (PD). Among various candidates, the cleaved extracellular domain known as Ecto-GPR37 has emerged as a measurable and potentially disease-specific indicator.

Ecto-GPR37 is produced through proteolytic cleavage of full-length GPR37, resulting in its release into the extracellular space. This truncated receptor fragment has been detected in brain tissue and cerebrospinal fluid (CSF), especially in PD cases (Mattila et al., 2016). The generation of Ecto-GPR37 is tightly linked to endoplasmic reticulum (ER) stress and misfolded protein responses, which are prominent in dopaminergic neurons affected by PD (Marazziti et al., 2009; Mattila et al., 2016). In a seminal study, Morató et al. (2021) demonstrated that Ecto-GPR37 levels are significantly elevated in the CSF of PD patients compared to healthy controls and patients with multiple system atrophy (MSA). Notably, this increase correlated with clinical severity and other neuronal injury biomarkers. Importantly, Ecto-GPR37 was undetectable in MSA, a disease that shares motor features with PD but lacks Lewy body pathology. This supports the idea that Ecto-GPR37 could serve as a disease-specific biomarker within the Lewy body spectrum (Morató et al., 2021). From a diagnostic standpoint, Ecto-GPR37 shows promise as a non-invasive fluid biomarker. It may complement existing α-synuclein assays and help differentiate PD from atypical Parkinsonian syndromes. Additionally, its detectability in early-stage disease opens the possibility for use in prodromal PD screening or disease progression monitoring (Argerich et al., 2024). Mechanistically, the presence of Ecto-GPR37 in CSF reflects both receptor misprocessing and disease-associated cellular stress. GPR37 is known to accumulate in the ER when misfolded, triggering cleavage and release of the ectodomain (Marazziti et al., 2009; Mattila et al., 2016). Therefore, its detection could serve not only as a diagnostic marker but also as a surrogate for underlying cellular dysfunction. However, several translational challenges remain. The molecular machinery regulating GPR37 cleavage remains poorly defined. Current detection techniques rely on immunoassays, which lack standardization and may not be sensitive enough for routine clinical use. Moreover, it is still unclear whether Ecto-GPR37 plays a pathophysiological role or simply reflects a cellular byproduct. Despite these limitations, Ecto-GPR37 stands out due to its selective expression in vulnerable neurons, its stress-induced cleavage, and its detectability in biofluids. These properties place it among a small group of GPCRs with biomarker potential. Continued validation in independent cohorts and longitudinal studies will be key to determining its utility in clinical practice (Morató et al., 2021; Qian et al., 2021).

## 6 Conclusion

G protein-coupled receptor 37 is a context-sensitive, multifunctional GPCR whose roles in the central nervous system span development, inflammation, neuroprotection, and disease. Over the past decade, substantial progress has been made in understanding its molecular identity, cell-type specificity, and diverse signaling mechanisms. From supporting oligodendrocyte maturation and synaptic regulation to modulating immune responses and pain perception, GPR37 functions as a molecular integrator of neural homeostasis. Disruption of GPR37 homeostasis–via aggregation, misfolding, or altered expression–has been implicated in a range of neuropathological conditions. In Parkinson’s disease, GPR37 misprocessing contributes to ER stress and dopaminergic neurodegeneration, while in multiple sclerosis and stroke, its dysregulation alters myelin repair and neurogenic responses. Moreover, GPR37’s involvement in autism spectrum disorder and glioma highlights its dual role in both neural development and oncogenesis, expanding its functional landscape beyond traditional neurodegenerative contexts.

Despite these insights, several critical knowledge gaps remain. The physiological relevance and disease-stage specificity of endogenous ligands such as TX14A, NPD1, and OCN are not fully established. The precise signaling cascades downstream of GPR37 activation differ across tissue and disease settings, complicating pharmacological targeting. Additionally, challenges persist in distinguishing the roles of GPR37 from its homolog GPR37L1, particularly in glial cells. Translationally, the cleaved extracellular fragment Ecto-GPR37 shows promise as a fluid biomarker in Parkinson’s disease, though standardization and longitudinal validation are required. Therapeutically, both ligand-based and genetic interventions have shown potential in modulating GPR37 activity in preclinical models. These early findings lay the groundwork for targeting GPR37 in future clinical interventions, particularly in disorders involving myelin deficits, neuroinflammation, or proteostasis dysregulation. Moving forward, future studies must aim to clarify ligand-receptor specificity, resolve spatiotemporal expression dynamics across disease stages, and leverage advanced tools such as conditional knockouts, spatial transcriptomics, and human-derived models. Further investigation into the crosstalk between GPR37 signaling and key pathways such as PI3K/Akt, MAPK/ERK, and ER stress will be vital for understanding its context-specific functions.

In conclusion, GPR37 represents both a molecular sensor of neurological stress and a modifiable node for therapeutic intervention. Its context-dependent functionality, pathological involvement, and pharmacological accessibility collectively position GPR37 as a compelling candidate in the landscape of CNS-targeted strategies.
